# Remarks on the Best Methods of Promoting Post-Partum Uterine Contractions

**Published:** 1877-08

**Authors:** George B. Fundenberg

**Affiliations:** Cumberland, Md.


Remarks on the Best Methods of Promoting Post-Partum Uterine Contractions.—*By George B. Fundenberg, M.D., Cumberland, Md.*—Expression of the placenta was first recommended by Crede, and other German writers, who taught that the placenta should never be removed by traction upon the cord, but by seizing the uterus through the abdominal walls and squeezing out its contents. This was certainly a step in advance, and Playfair made another, even more important, move in the right direction, when he insisted that, "in every labor, however normal, the practitioner should never remove his hand from the uterus after the birth of the child, until the placenta is expelled," and that he should "keep up continuous uterine contraction 'for at least half an hour after delivery; not necessarly by friction on the fundus, but by simply grasping the womb with the palm of the hand, and thus preventing undue relaxation." He also recommends the administration of ergot twenty minutes before and twenty minutes after delivery.

Playfair insists upon the extreme value of this proceeding in the prevention of post-partum hemorrhrge. In addition to this, I wish to call your attention to the many other advantages of this method, and especially to its value in the prevention of that form of septicæmia which arises from auto-infection. And as septicæmia is more frequent than dangerous post-partum hemorrhage, and more to be dreaded, because less under control, it becomes a question of prime importance whether the claim here made for this method of prevention can be sustained at the bedside. And as prevention is better than cure, any method that in a marked degree tends to prevent post-partum septic infection, must be hailed as an important advance in this department. If in addition to this, it will at the same time rescue the patient from the dangers of post-partum hemorrhage, while it nearly annihilates the after pains, and signally promotes uterine involution, its value must be greatly enhanced. It will be evident that the prophylactic value of this method will be most exhibited in those cases which arise from the retention of putrid material formed in the uterus and genital passages; but a firm contraction of the womb, and the consequent emptying of the vagina, may play an important part in the prevention of septic poisoning derived from without. Billroth lays it down as a law, that recent granulating wounds covered with pus cannot absorb. But is the placental disk from which the placenta has been recently separated, a wound? It certainly does not granulate, nor can the discharge from it be considered pus. It presents, however, when the uterus is relaxed, an open surface with numerous gaping sinuses, ready to imbibe whatever may be presented to their mouths, and this imbibition may be favored by the alternate relaxation and incomplete contraction of the uterus. Is it unreasonable to suppose that there may be a suction exercised by these sinuses under these circumstances? At least, it is certain that the laws which govern the diffusion of fluids must obtain here as elsewhere.

In the relaxed condition of the uterus and vagina after parturition, filled as they are with retained coagula and the detritus of broken down tissue, air is readily admitted, and putrefaction advances, by contiguity, from the external coagula to those more deeply seated. The temperature of the body, under these circumstances, favors rapid putrefactive changes, so that during the seventy-two hours succeeding delivery, enough of septic poison may be introduced into the system to produce the most deplorable results. Is it not possible, also, that under these circumstances, with coagula blocking up the os uteri and vagina, an inefficient contraction, and especially a partial one, may actually inject septic poison into the maternal sinuses? Whatever may be thought of these suggestions, every practitioner will recognize the value of that method which most certainly insures an enduring tonic contraction of the womb.

When we compare at the bedside the results following the old method, and the one I have been advocating, I believe every physician who has tried both will not hesitate long in delivering his verdict. How often do we witness cases in which, under the usual method of treatment, the placenta is without delay removed by traction, followed by the immediate application of the binder, and this succeeded by the administration of an opiate, which, if it have any other effect than annulling the perception of pain, most probably increases and makes more enduring the uterine relaxation. In many of these cases the after pains are more dreaded than those of labor, and the poor woman is worried and exhausted by ever recurring pains, until in many cases a state of uterine irritation is induced, bordering closely on inflammation, and occasionally terminating in it. How often have we seen patients treated in this manner discharging coagula even at the end of third week. It is easy to know how slowly and imperfectly involution must take place here, and how surely hyperplasia and displacement must occur. How different is the result when the placenta is carefully and leisurely expressed, the womb held in the grasp of the hand, and a tonic contraction insured, and when ergot given at the proper time comes in to take the place of the hand, and holds the uterus in its unrelaxing grasp. For that ergot establishes and keeps up a tonic contraction cannot be doubted. When a good preparation is given, in sufficient doses and at proper time, it rarely disappoints our expectations. When so timed that its full effect is obtained in half an hour after delivery, that is, as soon as the uterus is freed from the grasp of the hand, the full value of the method is illustrated. I do not wish to be understood as maintaining that this method will, in every case, produce these results, but that the cases are very few in which it fails; and even in these, in which the tendency to relaxation is unusually strong, the repeated use of ergot every four or six hours will give entirely satisfactory results. It is as a promoter of uterine condensation after parturition that ergot finds its most important therapeutical application. A short time ago I was called to a lady in her eighth confinement, in the last six of which I have been her attendant. This lady has been unusually subject to post-partum hemorrhages, and to severe and continued after pains, with all the unpleasant effects that follow. In her two previous accouchements she nearly lost her life by these floodings, and in the one previous to the last, I only succeeded in causing uterine contraction by introducing a piece of ice into the womb. In her late confinement I adopted the method recommended above, by the careful expression of the placenta, keeping the uterus in the grasp of the hand for thirty minutes, and the use of ergot before and after delivery, and although there was a rapid gush immediately after the delivery, the bleeding was easily controlled. The after pains were remarkably modified, and her recovery was unusually rapid; so much so, that she repeatedly inquired what could be the cause of such a difference between this and her former labors. And let it be remembered that this signal change occurred in the eighth pregnancy, when, other things being equal, the inertia of the womb should have been greater. I could cite a number of other cases equally significant, but, "*ex uno disce omnes*," they all teach the same lesson.

The ulterior results of insufficient uterine contraction are numerous. To this, in the large majority of cases, must be ascribed the hyperplasia, the menorrhagias, the leucorrheas, the displacements, and the other innumerable ills that follow in their wake, and that give such constant employment to the gynæcologist, and which so often constitute the opprobia of the profession.—*Med. and Surg. Reporter.*

